# Cost-effectiveness analysis of FOLFOX4 and sorafenib for the treatment of advanced hepatocellular carcinoma in China

**DOI:** 10.1186/s12962-018-0112-0

**Published:** 2018-08-04

**Authors:** Shukui Qin, Eliza Kruger, Seng Chuen Tan, Shuqun Cheng, Nanya Wang, Jun Liang

**Affiliations:** 1 Department of Medical Oncology, People’s Liberation Army Cancer Center, Eight One Hospital, Nanjing, China; 2 Economics and Outcomes, Real World Evidence, IMS Health, San Francisco, USA; 3 Economics and Outcomes, Real World Evidence, IMS Health, Singapore, Singapore; 4 Department of Hepatic Surgery, Shanghai Eastern Hepatobiliary Surgery Hospital, Shanghai, China; 5 Cancer Center, First Hospital of Jilin University, Jilin, China; 6 Department of Medical Oncology, Peking University Cancer Hospital, Peking University International Hospital, No. 1 Life Garden Road, Zhongguancun Life Science Park, Changping District, Beijing, 102206 China

**Keywords:** Cost-effectiveness analysis, FOLFOX4, Sorafenib, Advanced hepatocellular carcinoma, China

## Abstract

**Objectives:**

Hepatocellular carcinoma (HCC) is a leading cause of cancer-related deaths globally. In China, sorafenib and oxaliplatin plus infusional-fluorouracil/leucovorin (FOLFOX4) are approved for the systemic treatment of advanced HCC. This study compared the cost-effectiveness of these therapies from a healthcare system perspective and a patient perspectives.

**Methods:**

A Markov model was constructed using overall and progression-free survival rates and adverse event (AE) rate from two randomized controlled studies of advanced HCC patients from Asia: EACH for FOLFOX4 and ORIENTAL for sorafenib. The patients in the Markov model were followed until death, the length of each Markov cycle was 1 month, and the survival was adjusted for quality-adjusted life years (QALYs). Direct medical costs included costs of therapies, AE treatment, general ward and tests. Costs were derived from published sources, interviews with oncologists and hospital data from China. One-way and probabilistic sensitivity analyses (PSA) were performed to test the robustness of the results.

**Results:**

From the healthcare system perspective, FOLFOX4 dominated sorafenib with lower therapy costs (FOLFOX4: US$ 6972; sorafenib: US$ 12,289), lower direct medical costs (FOLFOX4: US$ 8428; sorafenib: US$ 12,798), and higher QALYs (FOLFOX4: 0.42; sorafenib: 0.38) per patient. This result was robust according to comprehensive one-way sensitivity analyses. According to the PSA, at the cost-effectiveness threshold for China (3 × GDP, US$ 22,073), FOLFOX4 should be chosen in 63.9% of simulations. From the patient perspective, FOLFOX4 also dominated sorafenib.

**Conclusions:**

The study results indicate that FOLFOX4 dominates sorafenib because it appears to provide higher effectiveness with significantly lower costs in treating Chinese advanced HCC patients.

**Electronic supplementary material:**

The online version of this article (10.1186/s12962-018-0112-0) contains supplementary material, which is available to authorized users.

## Background

Hepatocellular carcinoma (HCC) is a leading cause of cancer-related deaths globally. Over half of all global liver cancer cases occur in China [1], which has one of the highest incidences of HCC in the world at 34.4 and 12.2 for males and females, respectively, per 100,000 population [1]. Over 394,000 cases of liver cancer are diagnosed annually in China, resulting in 383,000 deaths and a mortality rate of over 97% [1].

HCC is known to be highly refractory to conventional systemic chemotherapy because of its heterogeneity and multiple etiologies [2]. Hepatectomy and liver transplantation are potentially curative treatment options in the early stage of HCC [3]. However, a large proportion of patients with HCC present with locally advanced or metastatic disease, at which point they are ineligible for curative treatments [4]. Systemic chemotherapy is a treatment option for patients with advanced or metastatic HCC, but the survival benefits of chemotherapy are usually minimal [3, 5]. The first approved systemic agent to show a survival benefit in patients with advanced disease was sorafenib, a multikinase inhibitor that inhibits tumor blood vessel development and tumor cell proliferation [6]. Sorafenib was the first therapy to be approved by the US Food and Drug Administration (FDA) to treat advanced HCC in 2007 and subsequently received regulatory approval for this indication in China in 2008. A double-blind placebo-controlled phase 3 trial of sorafenib in Europe, North America, South America, and Australia (SHARP study) found that the median overall survival was significantly greater in the sorafenib arm, 10.7 months, versus 7.9 months in the placebo arm [7]. The Global Investigation of therapeutic DEcisions in hepatocellular carcinoma and Of its treatment with sorafeNib (GIDEON), a non-interventional surveillance study, evaluated the safety and efficacy of sorafenib in patients with unresectable HCC in real-world practice. The safety analysis for Chinese subgroup showed that the median OS for Child–Pugh A was 10.7 months. However, a random double-blind placebo-controlled trial in Asia that included mainland China, Taiwan, and Korea (ORIENTAL study) showed that the median survival was significantly lower than in the SHARP study, with rates of 6.5 months and 4.2 months for the sorafenib and placebo arms, respectively [8].

Oxaliplatin (Eloxatin, Sanofi-Aventis) plus infusional-fluorouracil (FU) and leucovorin (LV) (FOLFOX4) was approved for the systemic treatment of advanced HCC patients in China in 2013. Oxaliplatin is a water-soluble platinum-based cytotoxic drug that prevents DNA replication by cross-linking DNA [9]. In a prospective, international, multicenter, open-label, randomized, phase III study of FOLFOX4 versus doxorubicin (DOX) in patients with advanced HCC from Asia including mainland China, Taiwan, Korea, and Thailand (EACH study), treatment with FOLFOX4 was found to improve overall survival (6.40 months versus 4.97 months) and progression-free survival (2.93 months versus 1.77 months) compared to treatment with DOX, while the Chinese sub-group analysis of EACH study showed that the overall survival was 5.9 months versus 4.3 months and the progression-free survival was 2.4 months versus 1.7 months, respectively [10, 11].

Cost-effectiveness evaluations have become an increasingly important component of health technology assessments. China has a large population of HCC patients and limited healthcare resources; therefore, achieving population health outcomes in a cost-effective manner is crucial. However, the cost-effectiveness of FOLFOX4 and sorafenib has not been evaluated. Therefore, the objective of this study was to compare the cost-effectiveness of FOLFOX4 and sorafenib for the treatment of patients with advanced HCC in China.

## Methods

### Model overview

A decision-analytic Markov model was constructed to simulate the disease process of advanced HCC and to estimate the comparative costs and effectiveness of FOLFOX4 and sorafenib in patients in China.

Patients who had advanced or metastatic hepatocellular carcinoma and who were ineligible for curative resection or local treatment were included in the model. Based on the disease progress of the patients, we defined three health states: progression-free survival (PFS), progressed disease (PD) and death in the Markov model. The Markov model began with 1000 patients. A patient was in one of these three states at a given time. A patient could remain in PFS (or PD) or progress to PD (or die) during each Markov cycle, and the patient sample was followed until death. The length of each Markov cycle was 1 month, and survival was adjusted for quality of life based on specific utilities. Direct medical costs were considered, including the costs associated with the respective therapies, tests, general ward and treatment of adverse events (AEs). All costs and effects were discounted by 5%, consistent with Chinese pharmacoeconomic guidelines [12]. The model development and data analysis were conducted using Microsoft^®^ Excel 2013 (Microsoft, Redmond, WA, USA).

### Efficacy and safety input

Randomized controlled trials (RCTs) were available comparing Oxaliplatin versus doxorubicin, sorafenib versus placebo, sorafenib versus sunitinib. FOLFOX4 and sorafenib have not been directly compared in a RCT. Moreover, the existing RCTs of FOLFOX4 and sorafenib do not have a shared comparison arm, making indirect analysis impossible. Two studies that investigated the efficacy of the respective therapies in the treatment of advanced HCC in Asian populations were identified: EACH for FOLFOX4 and ORIENTAL for sorafenib. Clinical efficacy inputs for the model were derived from the respective RCTs.

The ORIENTAL trial was a placebo-controlled, double-blind RCT that investigated the efficacy and safety of sorafenib in patients in the Asia–Pacific region [8]. A total of 271 patients from China, South Korea and Taiwan were randomized in a 2:1 ratio into sorafenib and placebo groups. Patients were considered eligible if they had been diagnosed with advanced HCC, had not received previous systemic therapy and had a Child–Pugh liver function class of A. More information on inclusion and exclusion criteria is available elsewhere [8]. Subgroup analyses of Chinese patients were not available.

The EACH trial was a randomized, multicenter, open-label study of oxaliplatin plus FU/LV (FOLFOX4) versus doxorubicin (DOX) for advanced HCC in Asian patients [11, 13]. A total of 371 patients aged 18–75 years who had been diagnosed with locally advanced or metastatic HCC and were ineligible for curative resection or local treatment were recruited from mainland China, Taiwan, Korea and Thailand. Patients with Child–Pugh class C liver function were excluded from the trial. A primary paper reported the results for all patients [13], while a secondary paper reported a subgroup analysis of Chinese patients (n = 279) [11]. All patients were followed until death in both studies.

Three HCC oncologists in China performed a blinded review of the baseline characteristics and inclusion/exclusion criteria and agreed that the characteristics of the patients who had been included in the two respective trials were comparable (Table 1). Inclusion/exclusion criteria for the respective studies are available in Additional file 1.

**Table 1:** Comparison of baseline characteristics

	FOLFOX4 (EACH)		Sorafenib (ORIENTAL)	
	n	%	n	%
N	184		150	
Age	49.53 (mean)		51 (median)	
Male	166	90.2	127	81.7
HBV infection	171	92.9	106	70.7
HCV infection	9	4.9	16	10.7
Child–Pugh stage				
A	163	88.6	146	97.3
B	21	11.4	4	2.7
BCLC stage				
B	39	21.2		
C	145	78.8	143	95.3
Extrahepatic spread	104	56.5	103	68.7

All the patients were followed until death in ORIENTAL All patients were followed until death in ORIENTAL study and only one patient left at the end of follow-up in EACH study. For the one patient alive in EACH study, we assume it will die in the next cycle. The numbers of surviving patients, patients with PD and deceased patients each month were obtained directly from the respective survival curves for sorafenib (ORIENTAL study) and FOLFOX4 (EACH study). Survival curves were translated into the proportion of surviving patients, assuming equal sized cohorts of 1000 patients. The incidence of AEs (greater than 10%, to be consistent with the ORIENTAL study where only AEs greater than 10% were reported) was extracted from the trial data [8, 13].

### Cost and utility Inputs

Treatment with FOLFOX4 and sorafenib in the model followed the respective trial designs, which were consistent with the recommended treatment in their drug instructions [14, 15]. The ORIENTAL study recommended that advanced HCC patients who were being treated with sorafenib take 400 mg twice daily for the duration of treatment [7]. The EACH study recommended that advanced HCC patients who were being treated with FOLFOX4 receive the following 48-h treatment cycle every 14 days (2.14 treatment cycles every 30 days): oxaliplatin 85 mg/m^2^ intravenously from hour 0 to 2 on day 1; LV 200 mg/m^2^ intravenously from hour 0 to 2 on days 1 and 2; and FU 400 mg/m2 intravenously (bolus) at hour 2, followed by 600 mg/m^2^ over 22 h on days 1 and 2 [10, 13]. The body surface area (BSA, cm^2^) was calculated as 0.0061 × height (cm) + 0.0128 × weight (kg) − 0.1529. We assumed an average height of 161.5 cm and an average weight of 61.8 kg, the same as an ordinary Chinese adult in 2012 [16]. Therefore, one treatment cycle of FOLFOX4 would require 138 mg of oxaliplatin, 649 mg of LV and 3247 mg of FU. Unit costs of drugs in FOLFOX4 therapy in China were extracted from the IMS China Hospital Pharmaceutical Audit (CHPA) database from the second quarter of 2015. The unit cost of sorafenib was calculated based on the negotiated price, as sorafenib was included in the national reimbursement drug list (NRDL) with a negotiated price in 2017.

In addition to the drug costs of FOLFOX4 and sorafenib therapy, interviews with three clinicians from three cities were conducted to collect the other medical resource use and cost data related to the two therapies. The medical resources related to only one treatment were analyzed; those commonly used in both treatments were not analyzed to estimate costs. As a result, in addition to the costs of therapies, the costs related to the general ward (for FOLFOX4 only), tests and treatment of AEs were included in the study. The recommended treatment and cost of AEs were obtained from interviews with three HCC clinicians in China. The cost estimated by the clinicians was averaged for use in the model. It was suggested by the clinicians that nausea and vomiting, as well as abnormal AST/ALT values, were treated simultaneously; consequently, we applied the cost to the highest incidence of either AE. Table 2 lists the costs of respective therapies, the general ward, tests and AE treatment, as well as the AE incidences for each therapy. The incidences of AEs in the trials were overall incidences during the trial period, so we allocated the incidence of AEs across the months proportionate to the number of surviving patients.

**Table 2:** Cost estimates and AE incidences

	Unit cost (US$)	Recommended dosage/frequency	Cost per month (30 days)
Drug therapy costs			
Sorafenib	1888 (60^a^ 200 mg)	400 mg twice per day	3777
FOLFOX4		≈ 2.14 treatment cycles per month	1865
Oxaliplatin	290 (50 mg)	138 mg per cycle	1716
5 FU	8 (400 mg)	3247 mg per cycle	132
LV	16 (1225 mg)	649 mg per cycle	18

	Unit cost (US$)	Incidence	
		Sorafenib	FOLFOX4
Other medical costs			
General ward^a^	8/day	/	5 days per cycle
Tests^b^	155/set	Once per month	Once per cycle
Adverse event	Cost/event		
Nausea + vomiting	65	11.4%	41.0%
Abnormal			
AST/ALT values	59	0.0%	31.7%
Alopecia	0	24.8%	0.0%
Anorexia	26	12.8%	26.8%
Bilirubin	349	0.0%	20.2%
Fatigue	3	20.1%	17.5%
Diarrhea	13	25.5%	15.9%
Sensory neuropathy	3	0.0%	15.3%
HFSR	4	45.0%	0.0%
Rash	7	20.1%	0.0%
Hypertension	37	18.8%	0.0%
Bone marrow suppression	79	0.0%	68.9%

^a^Only the hospitalization bed fee was counted, as other medical costs during hospitalization have been considered in the model

^b^Tests included abdominal ultrasound, MRI, hematological examination, and liver and kidney function. The tests with similar frequency for the two therapies were not included, e.g., TC tests with costs of US$ 310, which were performed once bi-monthly for each therapy

To be consistent with both trial designs, we assumed that the treatment ceased when the disease progressed. If treatment ceased within a period, whether due to death or PD, this occurred on average halfway through the month, and therefore half of the costs (general ward, tests, therapies and AEs treatment) were allocated.

Quality-adjusted life years (QALY), based on specific utilities of health states, were utilized to measure treatment outcomes. The utilities for each health state were 0.76 for PFS, 0.68 for PD and 0 for death, and the values were derived from Thomson et al. [17].

### Comparative cost-effectiveness

The costs and effectiveness of FOLFOX4 and sorafenib were compared using incremental cost-effectiveness ratios (ICERs). The World Health Organization (WHO) recommends that an intervention be classified as highly cost-effective if it costs less than the GDP per capita (US$ 7358 [18, 19]) and as cost-effective if it costs less than three times the GDP per capita (US$ 22,073 [20]). An intervention was considered to dominate if the QALYs that were gained were more than the alternative and the costs were less than the alternative.

### Perspectives

The base case was from the healthcare system perspective, in which the full costs of both universal health insurance-payment and patient-copayment were counted. In an additional scenario, we considered the patient perspective. In the patient perspective, we considered only the proportion of the patient-copayment for the cost of tests, general ward, AE treatment, sorafenib and FOLFOX4 drug therapy. With the integration of the medical insurance reimbursement policy for urban employees and urban and rural residents who were receiving oncology treatments, using Shanghai as an example, an average patient co-pay rate of 25% was applied for outpatients, mainly including drugs (sorafenib, FOLFOX4 therapy and AE treatment), and 15% for inpatients, including both general ward and tests [21].

### Sensitivity analyses

To test the model robustness, one-way and probabilistic sensitivity analyses (PSA) were conducted. Multiple one-way sensitivity analyses were performed for all parameters. Upper and lower inputs for one-way sensitivity analyses are available in Table 3. In one-way sensitivity analyses, the incremental net-health benefit (INHB) was calculated based on the following formula: INHB(λ) = (μ_E1 _− μ_E0_) − (μ_C1 _− μ_C0_)/λ = ΔE − ΔC/λ, where μ_Ci_ and μ_Ei_ were the cost and effectiveness of FOLFOX4 (i = 1) or sorafenib (i = 0), respectively [22], and λ was three times the GDP per capita of China in 2014.

**Table 3:** Sensitivity analysis parameters

Parameter	One-way sensitivity analysis		PSA	
	Base case value	Range	SD	Distribution
FOLFOX4 survival (PFS and OS)	100%	80–120%	20%	Normal
Sorafenib survival (PFS and OS)	100%	80–120%	20%	Normal
Sorafenib monthly cost	3777	3021–4532	1126	Gamma
FOLFOX4 monthly cost	1865	1492–2238	373	Gamma
Dosage per cycle Oxaliplatin	138	110–166	28	Normal
Dosage per cycle 5-FU	3247	2597–3896	649	Normal
Dosage per cycle L-FC	649	519–779	130	Normal
Utility PFS	0.76	0.61–0.91	0.152	Normal
Utility PD	0.68	0.54–0.82	0.136	Normal
General ward cost per cycle	39	19–78	8	Gamma
AE costs	100%	50/200%	20%	Gamma
Cost of HCC progression test (RMB)	155	78–310	93	Gamma
Proportion of FOLFOX4 general ward	100%	50%	N/A	N/A
Discount rate	5%	0–8%	N/A	N/A

PSA was performed to test the effect of uncertainty on the results for costs and effects. All model parameters were simultaneously and randomly sampled from a predetermined set of parametric distributions to generate 1000 estimates of the cost and QALYs for each intervention. The gamma distribution was selected for cost parameters, and the normal distribution was selected for probability, proportion and quality of life value parameters. The standard deviation (SD) was not available for our parameters. We assumed a standard deviation of 20% for all parameters, with the exception of 60% for non-therapy (general ward, AEs and tests) cost variables, which was mainly due to the limited number of key opinion leaders’ interviews conducted and the expectation that there would be a large variation in costs across China. For survival variables, we calculated the monthly transition probabilities (TPs) for PFS and OS and applied the variation to the transition probabilities simultaneously [23].

## Results

### Incremental cost-effectiveness ratio

From the healthcare system perspective, the cost of FOLFOX4 therapy was US$ 6972 per patient, compared to US$ 12,289 for sorafenib therapy (discounted). The costs related to the general ward, AEs and tests were higher for FOLFOX4, US$ 1456 per patient, versus US$ 509 per patient for sorafenib. After taking all costs into consideration, the total FOLFOX4 cost on average was US$ 8428 per patient, compared to US$ 12,798 for sorafenib; therefore, FOLFOX4 was US$ 4371 cheaper than sorafenib. At the same time, FOLFOX4 generated 0.42 QALYs versus 0.38 QALYs for sorafenib. As a result, FOLFOX4 dominated sorafenib. In other words, FOLFOX4 was both cheaper and more effective than the alternative. Table 4 displays the incremental cost and incremental effectiveness of FOLFOX4 compared to sorafenib.

**Table 4:** Incremental cost-effectiveness ratio (per patient)

	FOLFOX4	Sorafenib	Incremental
Discounted			
Drug costs	US$ 6972	US$ 12,289	− US$ 5317
General ward costs	US$ 145	US$ 0	US$ 145
AE costs	US$ 69	US$ 4	US$ 64
Tests	US$ 1242	US$ 504	US$ 738
Total costs	US$ 8428	US$ 12,798	− US$ 4371
QALY	0.42	0.38	0.034
			FOLFOX4 dominance

### One-way sensitivity analysis

According to one-way sensitivity analyses, the result was most sensitive to FOLFOX4 and sorafenib survival (PFS and OS) and the cost of sorafenib therapy. With the exception of the lower input for FOLFOX4 survival (PFS and OS) and the upper input for sorafenib survival (PFS and OS), FOLFOX4 dominated sorafenib. Nevertheless, for these two exceptional inputs, the ICER of sorafenib versus FOLFOX4 was over US$ 380 thousand per QALY.

**Fig. 1:**
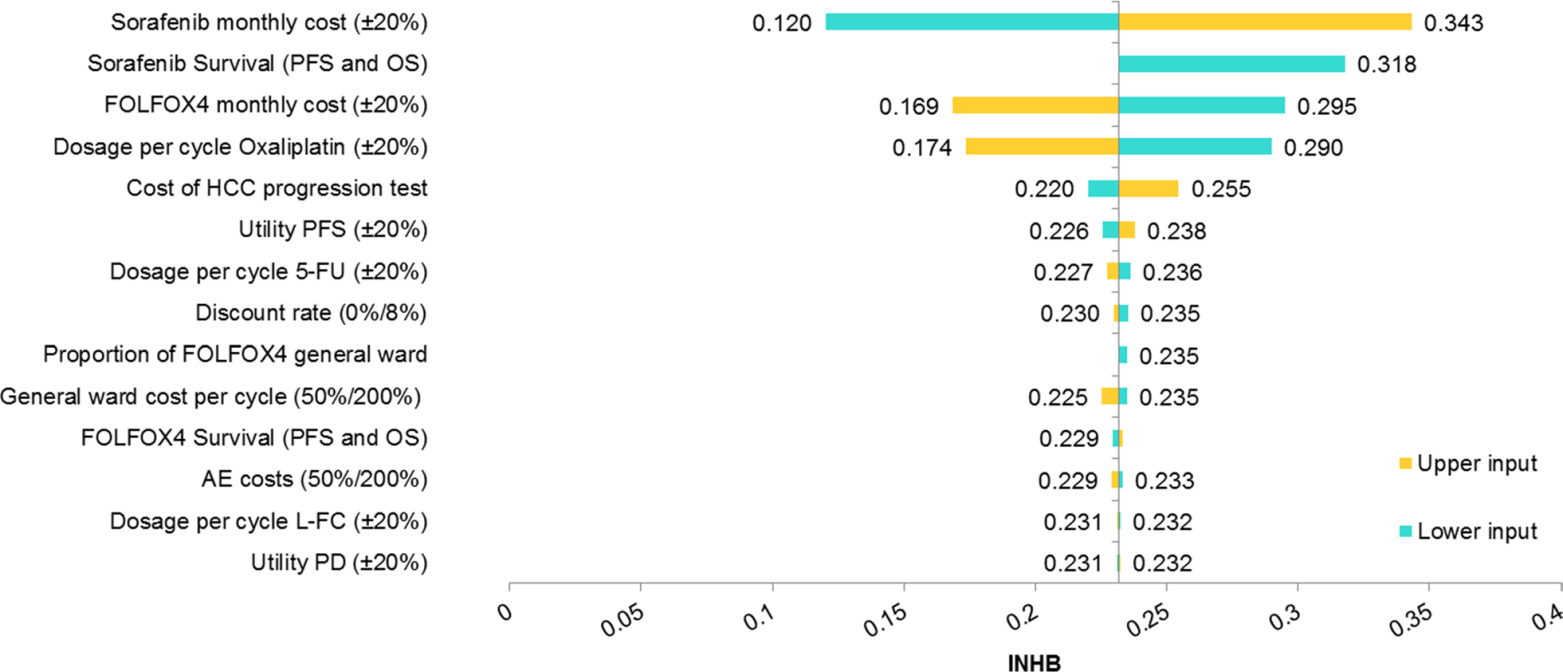
One-way sensitivity analyses (INHBs)

The INHB results are displayed in Fig. 1. The monthly costs of sorafenib therapy and sorafenib survival (PFS and OS) showed the largest impact on the INHBs. All INHBs that were generated were positive, indicating that FOLFOX4 was cost-effective compared to sorafenib for all given cases.

### Probabilistic sensitivity analysis

**Fig. 2:**
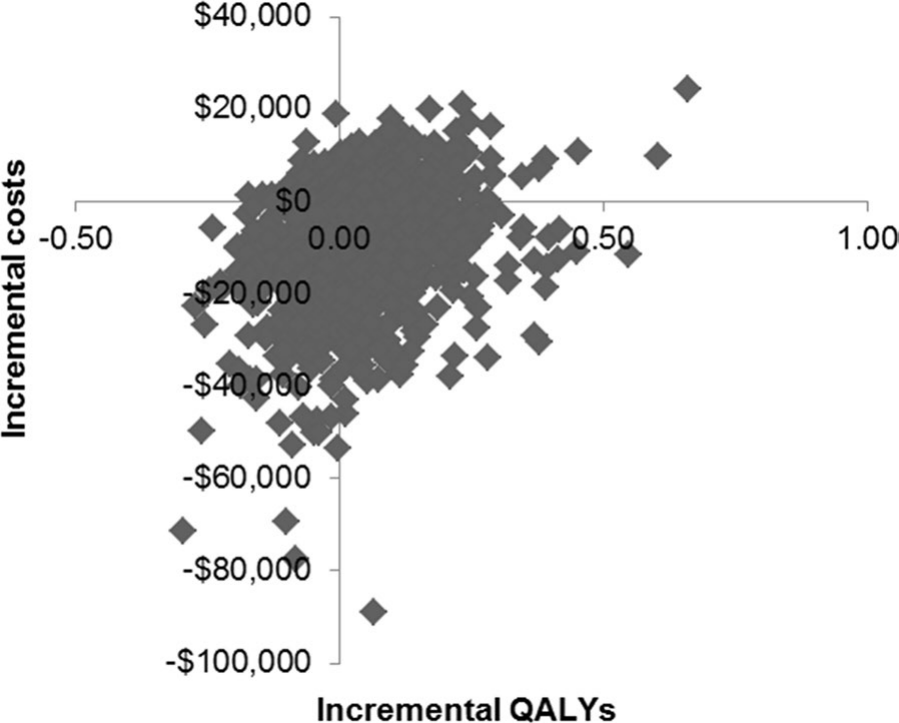
Probabilistic sensitivity analyses

In the PSA (Fig. 2), FOLFOX4 dominated sorafenib in 34.9% of simulations, while sorafenib dominated FOLFOX4 in 2.1% of simulations. At the cost-effectiveness threshold of 3 × GDP per capita, US$ 22,073, FOLFOX4 would be chosen in 63.9% of simulations.

### Patient perspective

From the patient perspective, which took into account the patient co-payments, the costs of both therapies were substantially reduced; however, the total costs per patient for FOLFOX4 (US$ 1395) remained lower than those for sorafenib (US$ 3200), and FOLFOX4 was more effective than sorafenib. Therefore, from the patient perspective, FOLFOX4 dominated sorafenib. The results and sensitivity analyses for the patient perspective scenario are available in Additional file 1.

## Discussion

Liver cancer is the second most common cancer in China, with one of the highest rates of mortality [1]. Hepatectomy and liver transplantation are potentially curative treatment options for patients with HCC. However, these treatments are appropriate only in the early stages of the disease. The first systemic agent to show a survival benefit in advanced HCC patients was sorafenib. Recently, FOLFOX4 was approved for the systemic treatment of patients with advanced HCC in China, providing an additional treatment option for advanced HCC patients. With no published studies having evaluated the cost-effectiveness of these two therapies in treating advanced HCC in China, a setting with scarce health resources, there is a need for economic evaluations to determine the best option for patients by considering both effectiveness and costs.

From the healthcare system perspective, the results from the model indicated that FOLFOX4 dominated sorafenib by providing higher effectiveness with significantly lower costs. The model estimated that, including the costs associated with therapies, tests, AE treatment and the general ward, FOLFOX4 could have a cost saving of US$ 4371 per patient. Meanwhile, this cost saving was associated with an improvement in effectiveness, with FOLFOX4 generating 0.42 QALYs per patient, compared to 0.38 QALYs for sorafenib. The robustness of the results was confirmed by one-way sensitivity analysis and PSA. In the one-way sensitivity analysis, FOLFOX4 was cost-effective for the tested value ranges and dominated in all parameters but two, indicating that sorafenib was far from being cost-effective (ICER over US$ 380 thousand per QALY compared to FOLFOX4). According to the results of the PSA, with the cost-effective threshold recommended by the WHO, FOLFOX4 was chosen in 63.9% of simulations, whereas sorafenib dominated in 2.1% of simulations.

Similar results were obtained for analyses from the patient perspective, which accounted for patient out-of-pocket costs, that out of the Chinese governmental basic medical insurance for sorafenib, oxaliplatin, hospitalization and drug costs. This analysis found that the patient self-paid proportion was substantially less for patients who had used FOLFOX4 than for those who had used sorafenib, at US$ 1395 and US$ 3200, respectively, per patient.

In addition, we calculated the BSA using the average height and weight value of healthy persons rather than HCC patients, who are likely to have lower body weight values, which would have overestimated the dosage and cost of FOLFOX4.

This study was the first to compare the cost-effectiveness of an oxaliplatin-based therapy, FOLFOX4, to sorafenib for the treatment of advanced HCC patients. Previous studies have compared the cost-effectiveness of sorafenib to best supportive care (BSC) [24–27] in advanced HCC patients who received palliative care and pain management. One recent study from China assessed the cost-effectiveness of sorafenib versus BSC using an analysis of retrospective safety and efficacy data [24]. The costs and effectiveness of sorafenib estimated by the authors were almost the same as those estimated in our study: US$ 19,149 and 0.45 QALYs per patient, compared to an estimated US$ 12,798 and 0.38 QALYs per patient in our model. The authors found that the ICER of sorafenib compared to BSC was US$ 101,399/QALY, almost five times greater than the WHO recommended willingness-to-pay threshold in China. Studies from Italy and the United States have also found that sorafenib versus BSC was not cost-effective [28, 29]. Moreover, NICE guidelines reported that sorafenib was not a cost-effective treatment compared to BSC for patients with advanced HCC using data from the SHARP trial, which reported significantly longer survival rates than the ORIENTAL trial data from the Asia–Pacific region [6].

Some potential specific drawbacks and limitations should be noted. First, the safety and efficacy data were derived from two separate RCTs. A blinded assessment of the baseline population summary statistics suggested that the characteristics of the patients in the respective trials were comparable, with the possibility that the patients enrolled in the EACH study (FOLFOX4) might have had more severe disease than those enrolled in the ORIENTAL study (sorafenib), which would make the model results conservative with regards to FOLFOX4. In addition, we performed several sensitivity analyses to test the impact of key parameters on the result. Nevertheless, we recommend that an RCT of FOLFOX4 versus sorafenib be conducted to provide direct comparative safety and effectiveness data. Second, the medical resource use and costs other than FOLFOX4 and sorafenib therapies and the treatments and costs of AEs were collected from interviews with three HCC oncologists in the Chinese clinical setting. Given the lack of specific recommendations for the relevant treatments and the variability in treatment and costs across China, it is difficult to determine how accurate and representative these costs are for all regions in China. We addressed this shortcoming in sensitivity analyses by varying the cost of AEs with a broader range—from half to double the estimated costs. Furthermore, we assumed that all patients who were being treated with FOLFOX4 needed to remain in the hospital, while it is possible that some patients may be treated as outpatients instead of inpatients, which was mentioned by the HCC clinicians in the interviews. In this case, we may have overstated the cost of the general ward for FOLFOX4, which would result in our estimates being conservative. Third, due to the limitation of data on the persistence of AEs and the patients’ quality of life with AEs and subsequent treatment, this study did not account for the detrimental impact of AEs on the quality of life of patients. This analysis was not conducted due to a lack of published literature on the duration and utilities for AEs. While this approach is consistent with cost-effectiveness analyses of sorafenib (for example, Camma [25], Zhang [24]), this represents one limitation of our study. In addition, similar to most economic evaluations, this study utilized utility scores from the published literature, which may vary among patients in different regions. To address this limitation, we examined the quality of life values for PFS and OS and found that the results were insensitive to this variable. Finally, because of several constraints specifically related to the context of Chinese clinical practice, the results of the present study must be considered strictly in the Chinese setting [30]. Nonetheless, because the results of this analysis reflect the clinical condition of patients with advanced HCC, which places a huge economic burden on the patient, family and healthcare system in China, we believe that the results can serve as important reference points for clinical decision makers in China.

## Conclusion

The present study revealed that FOLFOX4 showed higher effectiveness and lower costs than sorafenib in the treatment of advanced HCC patients in the Chinese setting, from both the healthcare system perspective and the patient self-paid perspective.

## Additional file

**Additional file 1: Table S1.** Inclusion and exclusion criteria of the EACH and ORIENTAL studies. **Table S2.** One-way sensitivity analysis, discounted (per patient), health care system perspective. **Table S3.** Patient scenario ICERs, discounted (per patient). **Table S4.** One-way sensitivity analyses (discounted), patient perspective. **Figure S1.** Incremental net health benefits, patient perspective. **Figure S2.** Probabilisitic sensitivity analysis, patient perspective.
