# Supplementary material for: Cost-effectiveness analysis of FOLFOX4 and sorafenib for the treatment of advanced hepatocellular carcinoma in China
Source: Cost Eff Resour Alloc. 2018 Aug 4;16:29. doi: 10.1186/s12962-018-0112-0 (PMC6076412; doi:10.1186/s12962-018-0112-0)

**Additional materials**

Additional file 1: Table S1 Inclusion and exclusion criteria of the EACH and ORIENTAL studies

| Criteria | FOLFOX4 (EACH) | Sorafenib (ORIENTAL) |
| --- | --- | --- |
| Age requirements | 18-75 years | 18+ |
| Invasive treatment | Ineligible for/unwilling to receive local invasive treatment | N/A |
| Previous treatment | Not received previous cancer treatment (except surgery) OR disease progression after previous interventional or local therapy with Karnofsky performance status 70+ | Not received previous systemic therapy OR target lesion increase in size by 25% + OR target lesion had not been treated with local therapy |
| How HCC diagnosed | Histologically, cytologically or clinically diagnosed unresectable HCC | Histologically or cytologically proved HCC |
| Lesions | Had at least 1 measurable lesion (>2cm on common CT, >1cm on spiral CT or MRI) | Had at least 1 measurable lesion (RECIST) |
| Life expectancy | 3 months + | 12 weeks+ |
| BCLC stage | B/C |  |
| EECOg PS |  | 0, 1 or 2 |
| Child-Pugh liver function | A/B | A |
| Platelet count |  | 60/10^9+ |
| Haemoglobin concentration |  | 85g/L + |
| Albumin concentration |  | 28 g/L + |
| Total bilirubin concentration |  | 51.3 umol/L or less |
| Alanine aminotransferase concentration |  | Less than 5 times upper limit of normal |

Additional file 2: Table S2 One-way sensitivity analysis, discounted (per patient), health care system perspective

|  |  |  | Lower output | | | Upper output | | |
| --- | --- | --- | --- | --- | --- | --- | --- | --- |
| **Parameter** | **Base case** | **Range** | **Incr. costs** | **Incr. QALY** | **ICER*** | **Incr. costs** | **Incr. QALY** | **ICER*** |
| Sorafenib Survival (PFS and OS) | 100% | 80%-120% | -US$ 2,048 | 0.085 |  | -US$ 7,806 | -0.036 | US$ 217,322 |
| FOLFOX4 Survival (PFS and OS) | 100% | 80%-120% | -US$ 5,831 | -0.031 | US$ 189,862 | - US$ 2,222 | 0.129 |  |
| Sorafenib monthly cost | 3,777 | 3,021-4,532 | -US$ 1,913 | 0.034 |  | -US$6,828 | 0.034 |  |
| Utility PFS | 0.76 | 0.61-0.91 | -US$ 4,371 | 0.028 |  | -US$ 4,371 | 0.040 |  |
| FOLFOX4 monthly cost | 1,865 | 1,492-2,238 | -US$ 5,765 | 0.034 |  | -US$ 2,976 | 0.034 |  |
| Dosage per cycle Oxaliplatin | 138 | 110-166 | -US$ 5,654 | 0.034 |  | -US$ 3,088 | 0.034 |  |
| Discount rate | 5% | 0% - 8% | -US$ 4,399 | 0.036 |  | -US$ 4,354 | 0.032 |  |
| Cost of HCC progression test | 155 | 78-310 | -US$ 4,118 | 0.034 |  | -US$ 4,875 | 0.034 |  |
| Utility PD | 0.68 | 0.54-0.82 | -US$ 4,371 | 0.033 |  | -US$ 4,371 | 0.034 |  |
| General ward cost per cycle | 39 | 19-78 | -US$ 4,443 | 0.034 |  | -US$ 4,226 | 0.034 |  |
| Dosage per cycle 5-FU | 3,247 | 2,597-3,896 | -US$ 4,469 | 0.034 |  | -US$ 4,272 | 0.034 |  |
| Proportion of FOLFOX4 general ward | 100% | 50% | -US$ 4,443 | 0.034 |  | -US$ 4,371 | 0.034 |  |
| AE costs | 100% | 50%- 200% | -US$ 4,403 | 0.034 |  | -US$ 4,307 | 0.034 |  |
| Dosage per cycle L-FC | 649 | 519-779 | -US$ 4,384 | 0.034 |  | -US$ 4,357 | 0.034 |  |

*Blank ICER – FOLFOX4 dominates

Additional file 1: Table S3 Patient scenario ICERs, discounted (per patient)

|  | **FOLFOX4** | **Sorafenib** | **Incremental** |
| --- | --- | --- | --- |
| **Discounted** |  |  |  |
| Drug costs | US$ 1,046 | US$ 3,072 | - US$ 2,027 |
| General ward costs | US$ 22 | ¥0 | US$ 22 |
| AE costs | US$ 17 | US$ 1 | US$ 16 |
| Tests | US$ 311 | US$ 126 | US$ 184 |
| Total costs | **US$ 1,395** | **US$ 3,200** | **-** **US$ 1,804** |
| QoL | **0.42** | **0.38** | **0.034** |
|  |  |  | **FOLFOX4 Dominance** |

Additional file 1: Table S4 One-way sensitivity analyses (discounted), patient perspective

|  |  |  | Lower output | | | Upper output | | |
| --- | --- | --- | --- | --- | --- | --- | --- | --- |
| **Parameter** | **Base case** | **Range** | **Incr. costs** | **Incr. QALY** | **ICER*** | **Incr. costs** | **Incr. QALY** | **ICER*** |
| Sorafenib Survival (PFS and OS) | 100% | 80%-120% | -US$ 1,224 | 0.085 |  | -US$ 2,663 | -0.036 | US$ 74,145 |
| FOLFOX4 Survival (PFS and OS) | 100% | 80%-120% | -US$ 2,045 | -0.031 | US$ 66,592 | -US$ 1,450 | 0.129 |  |
| Sorafenib monthly cost | 3,777 | 3,021-4,532 | -US$ 1,190 | 0.034 |  | -US$ 2,419 | 0.034 |  |
| Utility PFS | 0.76 | 0.61-0.91 | -US$ 1,804 | 0.028 |  | -US$ 1,804 | 0.040 |  |
| Discount rate | 138 | 110-166 | -US$ 1,820 | 0.036 |  | -US$ 1,796 | 0.032 |  |
| Patient co-payment - hospital | 15% | 10%-20% | -US$ 2,160 | 0.034 |  | -US$ 1,449 | 0.034 |  |
| FOLFOX4 monthly cost | 1,865 | 1,492-2,238 | -US$ 2,014 | 0.034 |  | -1,595 | 0.034 |  |
| Dosage per cycle Oxaliplatin | 138 | 110-166 | -US$ 1,997 | 0.034 |  | -US$ 1,612 | 0.034 |  |
| General ward cost per cycle | 39 | 19-78 | -US$ 1,815 | 0.034 |  | -US$ 1,783 | 0.034 |  |
| AE costs | 100% | 50%- 200% | -US$ 1,812 | 0.034 |  | -US$ 1,788 | 0.034 |  |
| Utility PD | 0.68 | 0.54-0.82 | -US$ 1,804 | 0.033 |  | -US$ 1,804 | 0.034 |  |
| Patient co-payment % - outpatient | 20% | 10%-30% | -US$ 1,230 | 0.034 |  | -US$ 2,379 | 0.034 |  |
| Dosage per cycle 5-FU | 3,247 | 2,597-3,896 | -US$ 1,819 | 0.034 |  | -US$ 1,790 | 0.034 |  |
| Dosage per cycle L-FC | 649 | 519-779 | -US$ 1,806 | 0.034 |  | -US$ 1,802 | 0.034 |  |
| General ward cost per cycle | 250 | 125 – 500 | -US$ 1,815 | 0.034 |  | -US$ 1,783 | 0.034 |  |
| Cost of HCC progression test | 155 | 78-310 | -US$ 1,741 | 0.034 |  | -US$ 1,930 | 0.034 |  |

*Blank ICER – FOLFOX4 dominates

Additional file 1: Figure S1 Incremental net health benefits, patient perspective


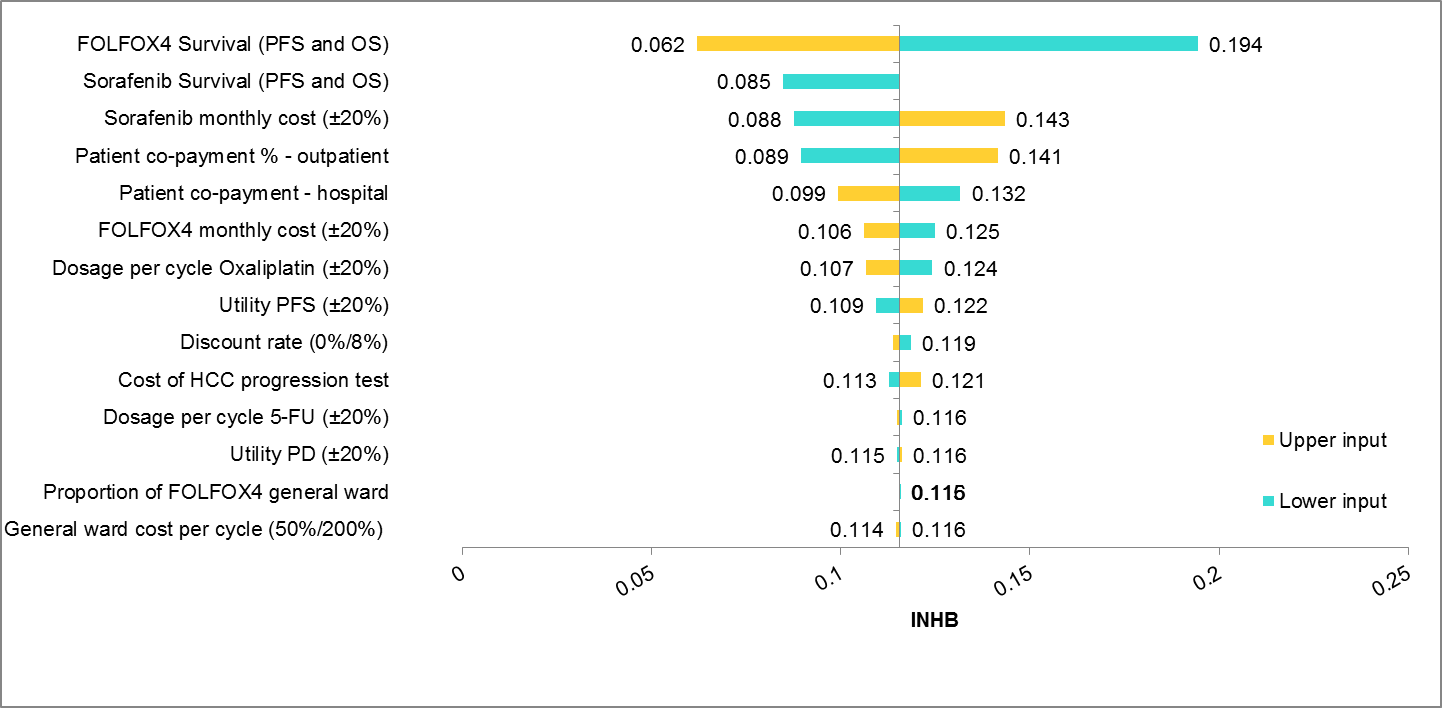


Additional file 1: Figure S2 Probabilisitic sensitivity analysis, patient perspective


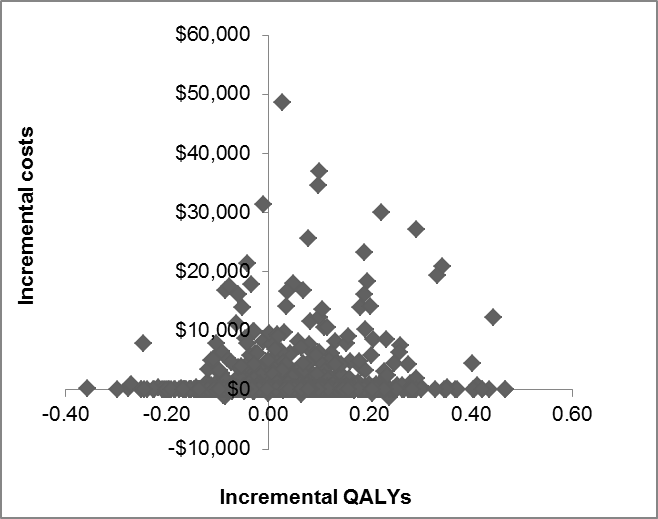

Supplement: Supplementary file 1 — Additional file 1: Table S1. Inclusion and exclusion criteria of the EACH and ORIENTAL studies. Table S2. One-way sensitivity analysis, discounted (per patient), health care system perspective. Table S3. Patient scenario ICERs, discounted (per patient). Table S4. One-way sensitivity analyses (discounted), patient perspective. Figure S1. Incremental net health benefits, patient perspective. Figure S2. Probabilisitic sensitivity analysis, patient perspective. [file 12962_2018_112_MOESM1_ESM.docx]
